# The Intestinal Roundworm *Ascaris suum* Releases Antimicrobial Factors Which Interfere With Bacterial Growth and Biofilm Formation

**DOI:** 10.3389/fcimb.2018.00271

**Published:** 2018-08-07

**Authors:** Ankur Midha, Katharina Janek, Agathe Niewienda, Petra Henklein, Sebastian Guenther, Diego O. Serra, Josephine Schlosser, Regine Hengge, Susanne Hartmann

**Affiliations:** ^1^Department of Veterinary Medicine, Institute of Immunology, Freie Universität Berlin, Berlin, Germany; ^2^Charité – Universitätsmedizin Berlin, Corporate Member of Freie Universität Berlin, Humboldt-Universität zu Berlin, and Berlin Institute of Health, Institute of Biochemistry, Shared Facility for Mass Spectrometry, Berlin, Germany; ^3^Charité – Universitätsmedizin Berlin, Corporate Member of Freie Universität Berlin, Humboldt-Universität zu Berlin, and Berlin Institute of Health, Institute of Biochemistry, Berlin, Germany; ^4^Department of Veterinary Medicine, Institute of Animal Hygiene and Environmental Health, Freie Universität Berlin, Berlin, Germany; ^5^Department of Pharmaceutical Biology, Institute of Pharmacy, Ernst-Moritz-Arndt-Universität Greifswald, Greifswald, Germany; ^6^Institute of Biology/Microbiology, Humboldt-Universität-zu-Berlin, Berlin, Germany

**Keywords:** intestinal nematode, ascariasis, helminth, microbiota, antimicrobial peptides, biofilm, lectin

## Abstract

Ascariasis is a widespread soil-transmitted helminth infection caused by the intestinal roundworm *Ascaris lumbricoides* in humans, and the closely related *Ascaris suum* in pigs. Progress has been made in understanding interactions between helminths and host immune cells, but less is known concerning the interactions of parasitic nematodes and the host microbiota. As the host microbiota represents the direct environment for intestinal helminths and thus a considerable challenge, we studied nematode products, including excretory-secretory products (ESP) and body fluid (BF), of *A. suum* to determine their antimicrobial activities. Antimicrobial activities against gram-positive and gram-negative bacterial strains were assessed by the radial diffusion assay, while effects on biofilm formation were assessed using the crystal violet static biofilm and macrocolony assays. In addition, bacterial neutralizing activity was studied by an agglutination assay. ESP from different *A. suum* life stages (*in vitro*-hatched L3, lung-stage L3, L4, and adult) as well as BF from adult males were analyzed by mass spectrometry. Several proteins and peptides with known and predicted roles in nematode immune defense were detected in ESP and BF samples, including members of *A. suum* antibacterial factors (ASABF) and cecropin antimicrobial peptide families, glycosyl hydrolase enzymes such as lysozyme, as well as c-type lectin domain-containing proteins. Native, unconcentrated nematode products from intestine-dwelling L4-stage larvae and adults displayed broad-spectrum antibacterial activity. Additionally, adult *A. suum* ESP interfered with biofilm formation by *Escherichia coli*, and caused bacterial agglutination. These results indicate that *A. suum* uses a variety of factors with broad-spectrum antibacterial activity to affirm itself within its microbe-rich environment in the gut.

## Introduction

Soil-transmitted helminth infections infect approximately 1.5 billion people worldwide (World Health Organization, 2017) as well as most companion, livestock, and wild animals (Eijck and Borgsteede, 2005; Nganga et al., 2008). The most prevalent helminth infection in people, Ascariasis, is caused by the intestinal roundworm *Ascaris lumbricoides* which infects approximately 800 million people (Brooker and Pullan, 2013) while the closely related *Ascaris suum* is commonly found in pigs raised for pork consumption (Dold and Holland, 2011; Thamsborg et al., 2013; Kreinoecker et al., 2017). The porcine host serves as a valuable infection model for humans for many diseases (Meurens et al., 2012), but particularly for Ascariasis, given the similarities between the human and pig intestinal tract and microbiota in comparison to that of mice (Heinritz et al., 2013) as well as the life cycles, genetic, and proteomic similarities of both *Ascaris* species (Leles et al., 2012; Xu et al., 2013; Shao et al., 2014). Infection begins with the ingestion of embryonated eggs containing L3-stage larvae which hatch in the small intestine before penetrating the intestinal wall of the cecum and colon to start their tissue migratory phase (Murrell et al., 1997). These L3-stage larvae then migrate through the liver before reaching the lungs by 6–8 days post-infection (Roepstorff et al., 1997). From the lungs, the larvae are coughed up and swallowed again, thereby reaching the small intestine where the nematodes will further develop into the L4 and adult stages and remain for approximately 1 year (Dold and Holland, 2011).

The small intestine hosts a microbiota, albeit at a lower density of microbes than that of the colon (Zoetendal et al., 2008; Isaacson and Kim, 2012; Sender et al., 2016). *A. suum* larvae invade host tissues in the distal small intestine, cecum, and proximal colon while adult worms reside in the small intestine; therefore, *A. suum* inhabits a microbial environment. Many studies have explored interactions between intestinal parasites and their hosts (Varyani et al., 2017), as well as hosts and their intestinal microbiota (Hooper et al., 2012); however, our understanding of how intestinal nematodes interact with the host microbiota is very limited. Recently, studies have linked various helminth infections to alterations in the host-intestinal microbiota (Zaiss and Harris, 2016). While host-immune factors and local metabolic factors have been implicated in shaping the microbiota, helminth components involved in the interaction with the microbial environment remain unexplored.

Studies in the free-living model nematode *Caenorhabditis elegans* suggest that these worms acquire an intestinal microbiota, distinct from their environments (Berg et al., 2016; Dirksen et al., 2016; Zhang et al., 2017). Though derived from environmental sources, the composition of the *C. elegans* microbiota was found to be selectively enriched and conserved across diverse sampling origins (Zhang et al., 2017). Additionally, certain microbes have been shown to support nematode growth and proliferation, while others pose infectious threats (Félix and Duveau, 2012; Samuel et al., 2016). Many laboratory-based studies have established *C. elegans* infection model systems with various bacterial pathogens (Couillault and Ewbank, 2002). Furthermore, other studies have also shown differential effects of biofilm-associated bacteria on *C. elegans* physiology (Tan and Darby, 2004; Begun et al., 2007; Smolentseva et al., 2017), demonstrating the diversity and importance of nematode-microbe interactions. Using these models, numerous details of the *C. elegans* antimicrobial defense response have identified detection mechanisms, transcription factors, and inducible effector molecules that form the nematode's innate immune system (Kim and Ewbank, 2015). In contrast in parasitic nematodes not much is known. Previous studies in *A. suum* have described induced transcription of members of two families of antimicrobial peptides (AMPs), *A. suum* antibacterial factors (ASABFs) and cecropins, in response to injection with heat-killed *Escherichia coli* (Pillai et al., 2003, 2005). In these studies, transcripts of some AMPs were also detected in the absence of an overt infectious challenge, suggesting that some defense molecules are produced constitutively. Homologs of ASABFs, called antibacterial factors, have also been described in *C. elegans* (Kato et al., 2002), as well as several other proteins and peptides involved in defense (Tarr, 2012).

Given the importance of interactions with bacteria for *C. elegans* physiology and longevity, as well as the absence of severe systemic inflammation of the host during Ascariasis despite migration of larvae originating in the intestine, we hypothesized a direct interaction of components of the intestinal parasitic nematode *A. suum* with the host gut-microbiota. Understanding the strategies that parasitic nematodes have evolved to control their microbial environments can provide insights into how the microbiota may be intentionally modified for therapeutic purposes, especially since nematodes do this without apparent detriment to their hosts. Herein we aimed to determine if *A. suum* nematodes release antimicrobial proteins and peptides in their excreted and secreted products (ESP) and whether or not these nematode products possess detectable antimicrobial activities.

## Materials and methods

### Ethics statement

All animal experiments were conducted in accordance with the principles of the European Convention for the Protection of Vertebrate Animals used for Experimental and other Scientific Purposes and ethical approval was obtained from the Landesamt für Gesundheit und Soziales Berlin, Germany (approval numbers H0288/15 and H0005/18).

### Parasite material

Adult *A. suum* worms were obtained from infected pigs at a local slaughterhouse. Upon retrieval, worms were separated by sex and washed several times in a balanced salt solution (BSS), recipe modified from Locke's solution (Chehayeb et al., 2014), containing antibiotics and used as culture media for adult worms (127 mM NaCl, 7.5 mM NaHCO_3_, 5 mM KCl, 1 mM CaCl_2_, 1 mM MgCl_2_, 200 U/mL penicillin, 200 μg/mL streptomycin, 50 μg/mL gentamicin, 2.5 μg/mL amphotericin B), then kept at 37°C with 5% CO_2_. Three to five adult worms were kept together in 300 mL of BSS. Media changes were completed daily by transferring worms to fresh bottles containing fresh BSS. To generate ESP for use in our experiments, worms were cultured in antibiotic-free BSS for several days with daily media changes. Spent media from the first 48 h were not used in microbiological assays. ESP were sterile filtered through a 0.22 μM vacuum-driven filter system and stored at −20°C until further use. For body fluid collection, adult worms were cultured in the absence of antibiotics as described for antibiotic-free ESP collection and body fluid was collected using the method of Chehayeb et al. (2014), sterile filtered using a 0.22 μM syringe-driven filter system and stored at −20°C until further use.

Third stage larvae were generated as previously described (Urban et al., 1981). Unembryonated *A. suum* eggs were collected from cultures of adult female worms, washed multiple times in water and placed in 0.1 N H_2_SO_4_ for 6–8 weeks at room temperature. Embryonation rates were assessed visually by light microscopy. Embryonated eggs were hatched using 5.25% hypochlorite treatment and incubation with slowly moving glass beads. Hatched third-stage larvae (L3) were cultured at a density of approximately 30,000 larvae/well of a 12-well tissue culture plate, in 1 mL of larval media [RPMI-1640 media (PAN Biotech, Aidenbach, Germany), 50 mM glucose, 200 U/mL penicillin, 200 μg/mL streptomycin, 50 μg/mL gentamicin, 2.5 μg/mL amphotericin B]. After 2 days in culture, worms were washed extensively with antibiotic-free media and then maintained in antibiotic-free larval media with media changes every 24 h for the first 2 days. Spent media from the first 48 h were discarded. Thereafter, supernatants were harvested every 48 h for 10–14 days, sterile filtered through a 0.22 μM syringe-driven filter system, and stored at −20°C until further use.

For tissue migrating larval stages, German Landrace piglets aged 8 weeks were orally infected with 12,000–15,000 embryonated *A. suum* eggs/pig. Pigs were sacrificed at 8 days post-infection for lung-stage larvae, and 16 days post-infection for L4-stage larvae. Lung-stage L3 larvae were retrieved as previously described with minor modifications (Slotved et al., 1997; Saeed et al., 2001). Briefly, harvested organs were ground using a hand-operated meat grinder. Ground organs were mixed with 0.9% NaCl to 300 mL and subsequently mixed with 300 mL of 2% agar solution which had been autoclaved and held at 45°C until use. The tissue-agar mixture was then poured into large glass petri dishes lined with plastic wrap and allowed to solidify, forming tissue gels. Tissue gels were wrapped in 200 μm woven synthetic mesh (Sefar, Edling, Germany), transferred to beakers with 0.9% NaCl, and incubated at 37°C for 3 h to allow worms to migrate into the saline solution. After 3 h, gels were removed and the remaining suspension transferred to Baermann funnels and allowed to sediment for 0.5–1 h. Worms were then collected and washed several times with larval media. Worms were cultured at 37°C with 5% CO_2_ with media changes every 24 h. Unfortunately, we were unable to retrieve antibiotic-free lung-stage larvae, so this material was excluded from microbiological assays.

For L4-stage larvae, pigs were sacrificed at 16 days post-infection and the distal small intestine and proximal cecum were removed. Intestinal contents were incubated in pre-warmed NaCl at 37°C for 3 h to allow larval migration away from host tissue and ingesta. This mixture was then poured over a Baermann funnel and allowed to sediment, then collected and washed extensively, and the worms cultured as described for L3-stage larvae, except with approximately 100 larvae per well of a 12-well tissue culture plate in 1 mL of larval media.

For use in the agglutination assay, adult *A. suum* ESP were concentrated using Vivaspin centrifugal concentrators with a 5 kDa molecular weight cut off (Sartorius, Göttingen, Germany) to a final protein concentration of 1 mg/mL. For LC-MS/MS analysis, ESP and BF samples were prepared as previously described (Eberle et al., 2015), with modifications. Oasis HLB Plus cartridges (Waters 186000132, Milford, USA) were rinsed with 2 mL of pure methanol, equilibrated with 3 mL of 0.2% formic acid, and loaded with either 5 mL of *A. suum* ESP or 3 mL of BF. Samples were washed with 5 mL of 0.2% formic acid then eluted with 1 mL of 30% acetonitrile/0.2% formic acid, then 1 mL of 60% acetonitrile/0.2% formic acid, and finally with 1 mL of 80% acetonitrile/0.2% formic acid. Eluates were pooled and dried in a centrifugal evaporator.

### Bacterial strains

The strains used to evaluate antibacterial activities of *A. suum* products in the radial diffusion assay included: *Escherichia coli* IMT19224, *Salmonella enterica* serovar Typhimurium (*S. typhimurium*) ATCC 14028, and *Staphylococcus aureus* IMT29828, all obtained from the strain collection of the Institute of Microbiology and Epizoonotics, Freie Universität Berlin. The strains used to assess the effects of *A. suum* ESP on biofilm formation included the biofilm forming *E. coli* K-12 strains AR3110 and AR115. *E. coli* IMT19224, AR3110, and AR115 were used to assess agglutinating activity of *A. suum* ESP. Strains were selected to include representative gram-negative and gram-positive bacterial strains which may model *A. suum*-microbe interactions or to elucidate anti-biofilm activities of *A. suum* ESP. *E. coli* IMT19224 is a sequence type 131 (ST131) strain; ST131 isolates are commonly multidrug resistant, producing extended-spectrum β-lactamases and resistant to fluoroquinolones (Nicolas-Chanoine et al., 2014). *E. coli* AR3110, derived from *E. coli* K-12 strain W3110 by correcting a single nucleotide polymorphism in the *bcs* operon, produces phosphoethanolamine-modified cellulose and amyloid curli fibers as predominant extracellular matrix components in macrocolony biofilms (Serra et al., 2013; Thongsomboon et al., 2018). *E. coli* AR115 was derived from AR3110 by deleting *wcaE*, a gene involved in colanic acid synthesis (Miajlovic et al., 2014).

### Radial diffusion assay

Antibacterial activities of ESP were assessed using the radial diffusion assay (Takemura et al., 1996). Overnight cultures were diluted 1:100 in Mueller-Hinton Broth (Carl Roth, Karlsruhe, Germany) and incubated at 37°C with shaking at 250 rpm until reaching an optical density of 0.3–0.4 at 600 nm. The bacteria were centrifuged at 880 x *g* for 10 min at 4°C, washed once with cold sodium phosphate buffer (100 mM, pH 7.4), and resuspended in cold sodium phosphate buffer. Bacteria were suspended in previously autoclaved, warm (50°C) underlay agar (10 mM sodium phosphate buffer, 1% (v/v) Mueller-Hinton broth, 1.5% (w/v) agar), at 4 × 10^5^ colony forming units per mL. 15 mL of underlay agar was poured into 120 mm square petri dishes and allowed to solidify. Using the blunt ends of P10 pipet tips, evenly spaced 5 mm wells were punched into the agar into which 5 μL of treatments and controls were added. Adult and larval growth media were included as negative controls. The *A. suum* AMP Cecropin P1 (Sigma-Aldrich, St. Louis, USA) was also included in the analysis. Plates were then incubated at 37°C for 3 h and then overlaid with 15 mL of overlay agar (4.2% (w/v) Mueller-Hinton broth, 1.5% (w/v) agar). The plates were incubated for 18 h at 37°C and the growth inhibition zones around each of the wells were measured. Antibacterial activity is herein represented as the diameter of the inhibition zone (mm) beyond the well.

### Crystal violet assay

The influence of *Ascaris* ESP on biofilm formation was assessed using the microtiter dish biofilm formation assay (O'Toole, 2011). The biofilm forming *E. coli* K-12 strains AR3110 and AR115 were grown overnight in liquid salt-free Luria-Bertani (LB) medium at 37°C. The overnight culture was diluted in 2X LB medium (9 × 10^8^ colony forming units per mL) for use in the biofilm assay. Hundred microliter of this bacterial suspension was used per well of a 96-well tissue-culture plate (Corning, New York, NY, USA) in replicates of four. The final volume per well was 200 μL with the remaining volume made up of controls and treatments at the concentrations indicated in the text. The plates were incubated for 24 h at 37°C. After incubation, cell suspensions were removed and the wells washed twice with phosphate buffered saline (pH 7.2) and stained for 15 min at room temperature with 0.1% (w/v) crystal violet solution (Sigma-Aldrich). The wells were then washed twice with distilled water and air-dried. For quantification, 125 μL of 30% acetic acid were added to each well and the plate incubated at room temperature for 15 min. The solubilized stain was transferred to a fresh flat-bottom 96-well plate and the absorbance read at 550 nm. Statistical analyses were performed using GraphPad Prism 7.0a to conduct 2-way ANOVA followed by Tukey's multiple comparison tests. *P-*values less than 0.05 were considered significant.

### Macrocolony biofilm assay

The influence of *Ascaris* ESP on the morphology of biofilms was assessed using the macrocolony biofilm model (Serra and Hengge, 2017). Experiments were carried out using the same strains as for the crystal violet biofilm formation assay. Cells were grown overnight in salt-free LB medium at 37°C. 5 μL of the overnight culture was spotted on salt-free LB agar plates containing Congo red 40 μg/mL and Coomassie brilliant blue 20 μg/mL. 35 mm petri dishes (Sarstedt, Nümbrecht, Germany) were used to grow one colony per plate. After autoclaving and cooling to 42°C, agar was prepared with controls and treatments at the indicated final concentrations. Colonies were incubated at 28°C for up to 5 days. Macrocolonies were visualized at 10X magnification with a Stemi 2000-C stereomicroscope (Zeiss, Oberkochen, Germany) and photographed with an AxioCamICC3 digital camera (Zeiss).

### Agglutination assay

Agglutination activity of nematode products was assessed as previously described (Gasmi et al., 2017), using *E. coli* IMT19224. Bacteria were collected at mid-logarithmic phase by centrifugation at 880 × g for 5 min, washed then resuspended in BSS at approximately 10^9^ cells/mL. 20 μL of bacteria were mixed with 20 μL of treatments in the presence and absence of 10 mM CaCl_2_ and incubated for 1 h at room temperature on a glass slide. Concanavalin A from *Canavalia ensiformis* (Con A) and Lectin from *Triticum vulgaris* (Wheat germ agglutinin; WGA, both from Sigma-Aldrich) were included as positive controls. Samples were then visualized and photographed at 40X magnification on a Leica DM750 microscope equipped with an ICC50HD digital camera (Leica Microsystems, Wetzlar, Germany).

### In-solution tryptic digestion and LC-MS/MS analysis

Dried protein samples were resuspended in 50 μL of 50 mM ammonium bicarbonate in 5:95 (v/v) acetonitrile/water (digestion buffer) and reduced with 8 μL of 45 mM dithiothreitol in digestion buffer at 60°C for 30 min. After cooling to room temperature 8 μL iodoacetamide solution (100 mM in digestion buffer) were applied and the sample was kept in the dark for 30 min. Subsequently the samples were diluted with 190 μL digestion buffer and digested with 0.15 μg trypsin at 37°C for 4 h. The reaction was stopped with 2.5 μL of 10% (v/v) trifluoroacetic acid in water. The samples were concentrated to approximately 50 μL and desalted with μC18-ZipTips (Millipore, Darmstadt, Germany), dried and reconstituted in 0.1% (v/v) trifluoroacetic acid in 2:98 (v/v) acetonitrile/water. LC-MS/MS analyses of peptides were performed on an Ultimate 3000 RSLCnano system online coupled to an Orbitrap Q Excative Plus mass spectrometer (Thermo Fisher Scientific). The system comprised a 75 μm i.d. × 250 mm nano LC column (Acclaim PepMap C18, 2 μm; 100 Å; Thermo Fisher Scientific). Mobile phase (A) was 0.1% formic acid in 2:98 (v/v) acetonitrile/water and (B) 0.1% formic acid in 80:20 (v/v) acetonitrile/water. The gradient was 3–40% B in 85 min. Full MS spectra (350–1,600 m/z) have been acquired at a resolution of 70.000 (FWHM) followed by a data-dependent MS/MS fragmentation of the top 10 precursor ions (resolution 17.500; 1+ charge state excluded, isolation window of 1.6 m/z, normalized collision energy of 27%). The maximum ion injection time for MS scans has been set to 50 ms and for MS/MS scans to 80 ms.

### Database searching and sequence analysis

Protein identifications were performed with Mascot software version 2.6.1 (Matrix Science Ltd., London, UK). Data were searched against an *A. suum* protein database from nematode.net (http://nematode.net/NN3_frontpage.cgi?navbar_selection=speciestable&subnav_selection=Ascaris_suum), 17,843 sequences, 2017_05), *A. suum* proteins from Uniprot (9,213 sequences, 2017_05), the antimicrobial peptide database (http://aps.unmc.edu/AP/main.php, 2,338 sequences; 2017_05), SwissProt (555,100 sequences, 2017_07) and a contaminant database (247 sequences). The following parameters were set: enzyme: trypsin/P with one missed cleavage, static modification: carbamidomethylation (C), variable modifications: oxidation (M) and pyro-glu (Q), mass tolerances for MS and MSMS: 5 ppm and 0.02 Da. Proteins were accepted as identified if at least two unique peptides with *p* < 0.01 were detected. Proteins identified only by one peptide were verified by comparison of their peptide fragment pattern with those of synthetic analogs. These reference peptides were synthesized in-house using Fmoc solid phase chemistry as previously described (Venken et al., 2011). In case of the common peptide (ISEGIAIAIQGGPR) of cecropin P1 and P2 an identification threshold of *p* < 0.00001 was set. Protein sequences were analyzed for the presence of classically secreted proteins containing signal peptides using SignalP 4.1 (Petersen et al., 2011) and for non-classically secreted proteins using SecretomeP 2.0 (Bendtsen et al., 2004).

## Results

### *Ascaris suum* ESP possess antibacterial activity

**Figure 1:**
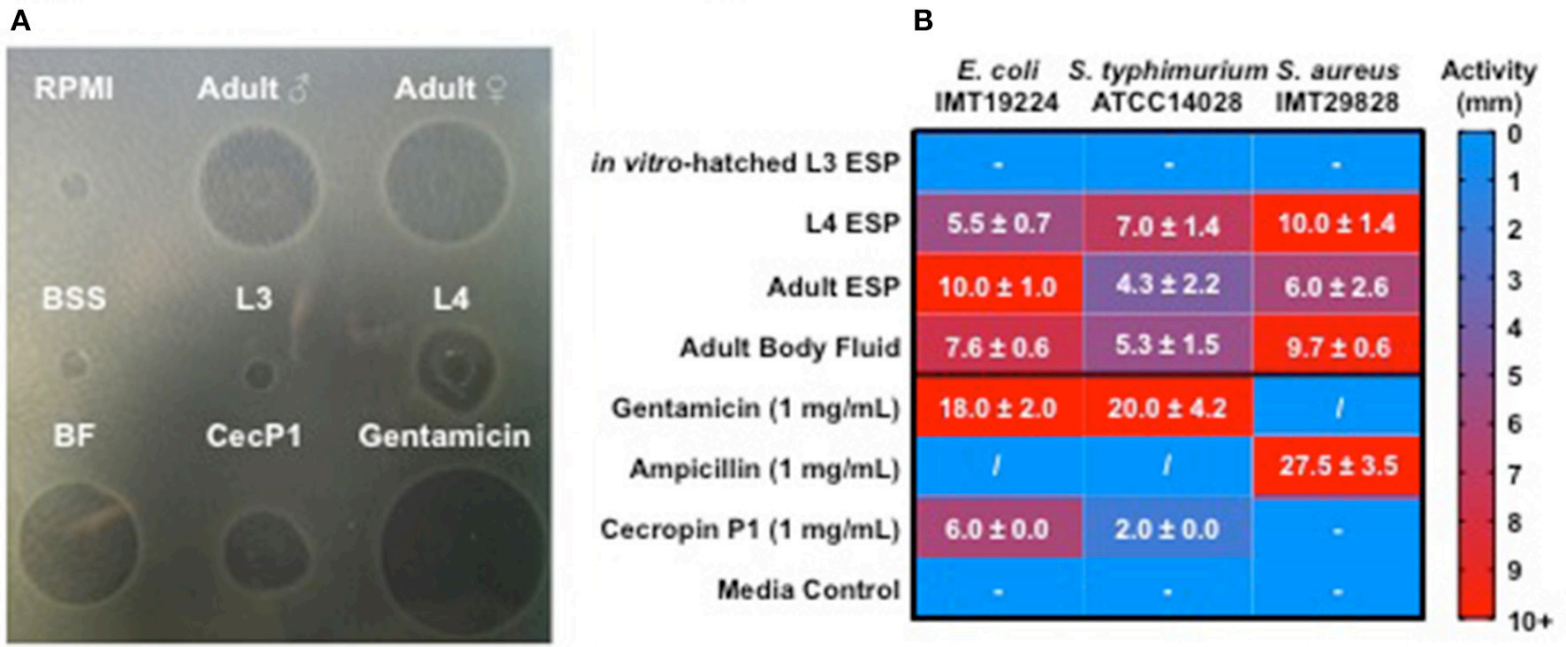
*Ascaris suum* excretory/secretory products and body fluid possess antimicrobial activity. Five microliter of nematode products were applied to agar plates with proliferating bacteria for 18 h at 37°C and growth inhibition zones measured in millimeters. *Ascaris* products tested include native excreted and secreted products (ESP) from adult worms kept in culture for 24 h, body fluid (BF) from adult males, native ESP from approximately 30,000 L3-stage larvae hatched *in vitro/*mL media, native ESP from approximately 100 L4-stage larvae/mL media, and a synthetic form of the *A. suum* antimicrobial peptide, cecropin P1. Larval (RPMI) and adult worm media (BSS) were included as controls. **(A)** Representative agar plate of a radial diffusion assay, with nematode products tested against *E. coli*. **(B)** Activity shown as diameter (mm) of inhibition zones on agar plates. Results are expressed as means ± standard deviations obtained from 2 to 3 independent experiments with multiple batches of *A. suum* products (L3 *n* = 3, L4 *n* = 2, adult ESP and body fluid n = 3). “−” represents no detected activity. “/” = not tested.

As intestinal parasitic nematodes inhabit a microbe rich environment, they are likely to experience microbial challenges while dwelling in the intestine. These challenges would need to be managed in order for the parasite to establish itself and survive during a long-term infection. We used the radial diffusion assay to test the antibacterial activity of native, unconcentrated secreted products (ESP) of different *A. suum* life stages and body fluid (BF) of adult male worms. The activities of nematode products against *E. coli* ST131 IMT19224, *S. aureus* IMT29828, and *S. typhimurium* ATCC 14028 were assessed. Adult ESP were obtained from 3 to 5 adult worms kept in 300 mL of culture medium (BSS), L3-stage material was harvested from the pooled supernatants of 30,000 larvae/well of a 12-well plate in 1 mL of larval media, while L4-stage material was harvested from the pooled supernatants of 100 larvae/well of a 12-well plate. BF was pooled from 5 adult males per batch. Treatments of ESP and BF were applied to proliferating bacteria and the resulting growth inhibition zones measured in comparison to BSS and larval culture media as controls (Figure 1). *Ascaris* ESP from *in vitro*-hatched L3-stage larvae resulted in no observable antibacterial activity. In contrast, ESP harvested from L4-stage larvae were very active, resulting in growth inhibition zones comparable to synthetic cecropin P1 against *E. coli*, and considerably more active than cecropin P1 against *S. typhimurium*. Interestingly, cecropin P1 had no detectable activity against *S. aureus*. Adult ESP were active against all strains tested and no considerable difference was detected between male and female ESP, thus they were considered together as “Adult ESP.” BF from adult males demonstrated activity comparable to that of L4-stage larval ESP. Thus, these results show that native parasite material harvested directly from *A. suum*, including ESP and BF, possess considerable antibacterial activity. ESP from the intestinal L4 and adult life stages were most active, whereas ESP from *in vitro*-hatched L3 larvae did not show antibacterial activity.

### *Ascaris suum* ESP impair bacterial biofilm formation

**Figure 2:**
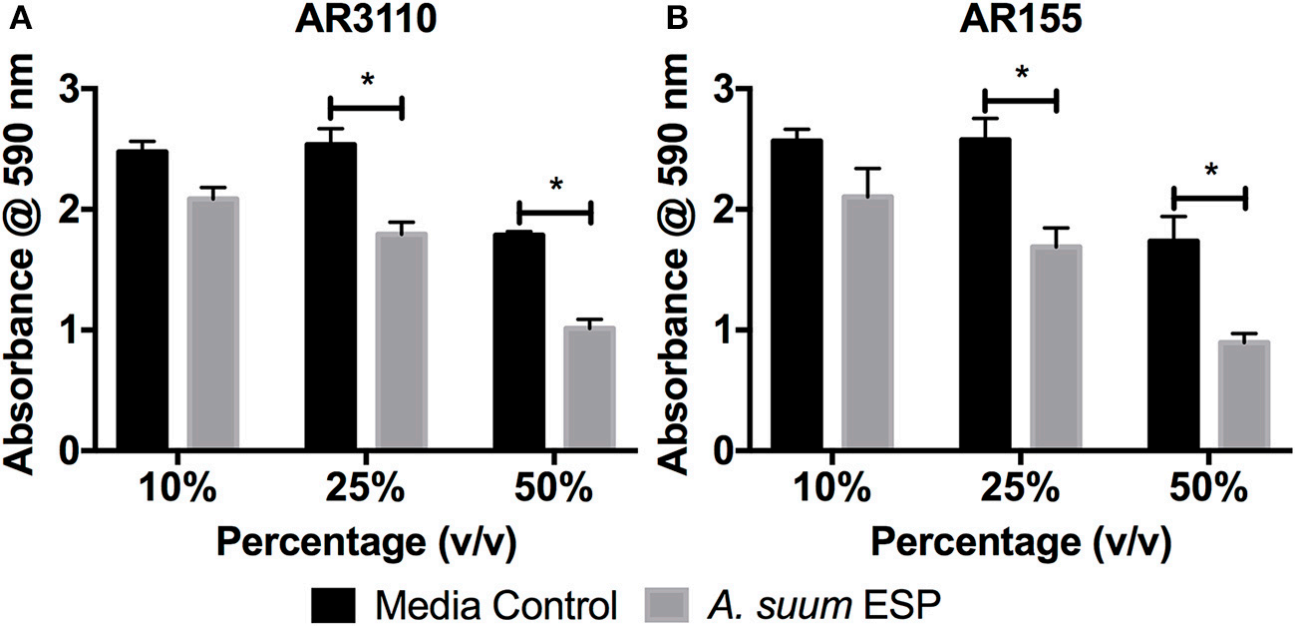
*Ascaris suum* excretory/secretory products decrease biomass of submerged biofilms. Biofilm forming *E. coli* K-12 strains **(A)** AR3110 and **(B)** AR115 (a *wcaE* derivative of AR3110) were grown in 96-well cell culture plates in salt-free LB medium for 18 h at 37°C in the presence of adult *A. suum* excreted/secreted products (ESP) or adult worm media (BSS) as a control. Treatment doses were added as a percentage (v/v) of final culture volume (total = 200 μL per well). Results represented as the mean of three independent experiments ± SEM. Significance determined by 2-way ANOVA with Tukey's multiple comparison tests, *p < 0.05.

Many species of bacteria live in communities known as biofilms in which cells are embedded in an extracellular matrix of self-produced polymers. In addition to representing the preferred lifestyle in nature for many bacteria, biofilms are often of medical relevance for infectious diseases (Hall-Stoodley et al., 2004; Flemming et al., 2016). In the case of free-living *C. elegans* nematodes, biofilms have been shown to be harmful, contributing to the pathogenicity of *Staphylococcus epidermidis* against the worm (Begun et al., 2007), whereas biofilm formation by *Bacillus subtilis* enhances nematode stress resistance (Smolentseva et al., 2017). Therefore, as biofilms may also be of importance to intestinal nematodes, we evaluated the effects of *A. suum* ESP on biofilm formation using the submerged biofilm model (O'Toole, 2011) and the macrocolony biofilm model (Serra et al., 2013). We used the biofilm-forming *E. coli* K12 strain AR3110, which is a W3110 derivative with restored capacity to produce phosphoethanolamine-modified cellulose (pEtN-cellulose). AR3110 produce pEtN-cellulose along with amyloid curli fibers as key components of the extracellular matrix in biofilms (Serra et al., 2013; Thongsomboon et al., 2018). PEtN-cellulose production has been restored by repairing a single nucleotide polymorphism that resulted in a stop codon in the *bcs* operon (Serra et al., 2013). As adult worms can survive for approximately 1 year in the intestine, growing between 15 and 25 cm in length (Dold and Holland, 2011), they may present surfaces on which biofilms can form in the small intestine. Hence, we used adult material to study the impact of *A. suum* ESP on biofilm formation. In the submerged biofilm assay, bacterial suspensions were mixed with *A. suum* ESP in a volume-dependent manner as indicated and inoculated into the wells of flat-bottom 96-well tissue culture plates and grown for 18 h at 37°C. The same concentrations of adult culture media, BSS, were used as media controls. Biofilm formation was assessed by crystal violet staining of the biomass that had formed on the submerged wall and bottom of the wells, thereby staining bacterial cells as well as extracellular matrix components. *A. suum* adult ESP demonstrate a dose-dependent inhibition of bacterial biofilm formation for both strains tested, in comparison to control (Figure 2).

**Figure 3:**
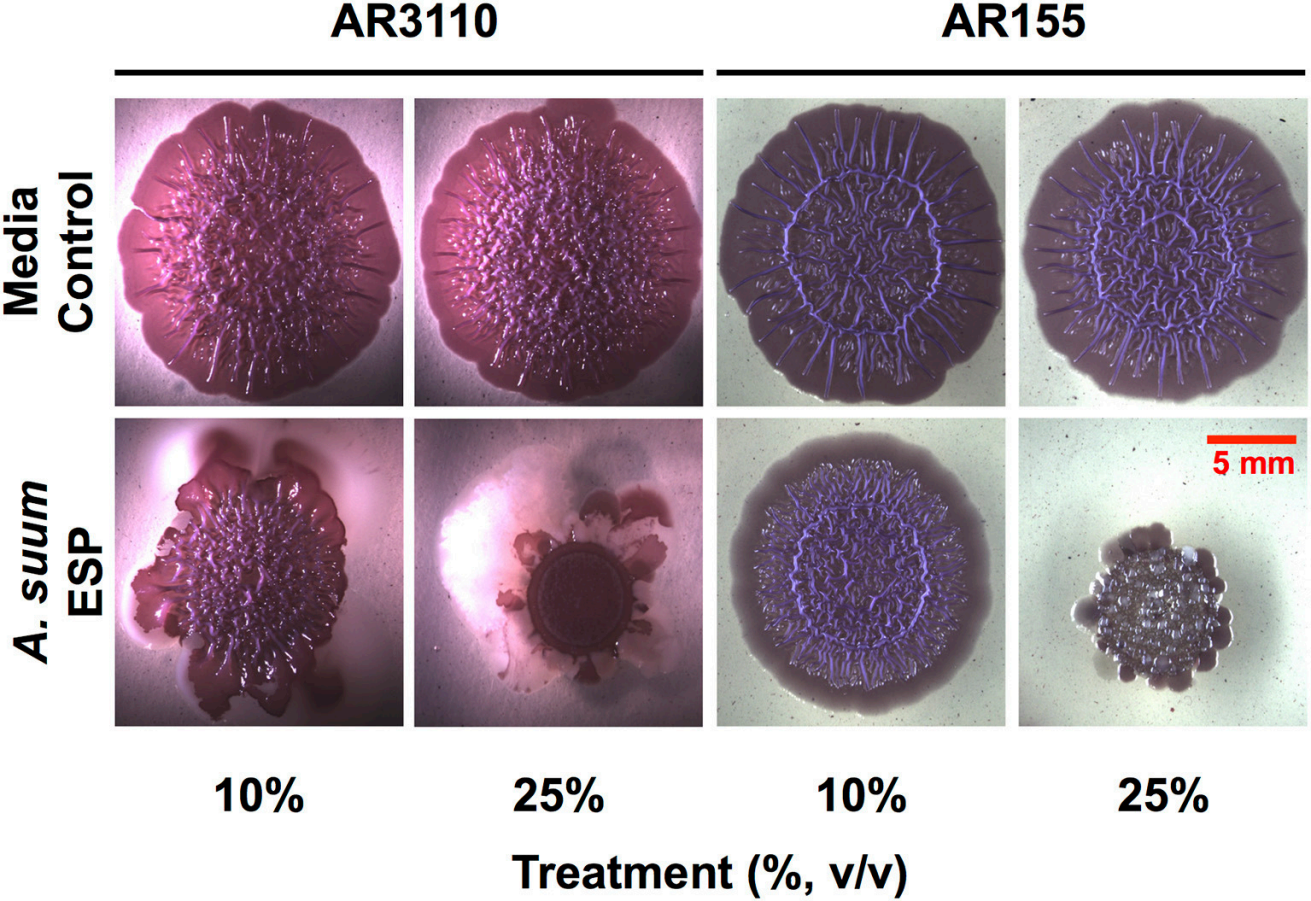
*Ascaris suum* excretory/secretory products impair macrocolony biofilm formation. Five microliter of bacterial suspensions grown overnight were spotted on salt-free LB agar plates supplemented with Congo red, a dye that acs as an extracellular matrix indicator staining both pEtN-cellulose and curli fibers, as well as Coomassie brilliant blue and infused with either adult worm media (media control) or adult *A. suum* excreted/secreted products (ESP). Inoculated plates were then grown for up to 5 days at 28°C. Images shown here correspond to 5-days-old macrocolony biofilms of *E. coli* AR3110 and AR155 strains treated or untreated with *A. suum* ESP at two different concentrations.

In the macrocolony biofilm assay, a dose-dependent disruption of colony growth was observed (Figure 3). With the AR3110 strain, the overall size of the resultant colony was decreased in the presence of *A. suum* adult ESP. Importantly, with 25% of ESP, *E. coli* responded to the treatment by producing large amounts of a white viscous substance (white shiny colony sectors; Figure 3). Since this substance was not formed in the *wcaE* mutant AR155, it can be ascribed to colanic acid, a mucoid exopolysaccharide that is typically produced in response to cell envelope stress and can confer resistance to antimicrobial insults and desiccation (Detweiler et al., 2003; Laubacher and Ades, 2008). This indicates that at least some of the ESP constituents act on the *E. coli* cell envelope, causing stress. However, also for the colanic acid-free mutant AR155, growth was not completely abolished suggesting that bacteria still resist the treatment by alternative mechanisms other than the production of colanic acid. Thus, these results show that the bacteria are able to adapt and survive to *A. suum* adult ESP, albeit while displaying signs of considerable stress. Notably, treatment with *A. suum* adult ESP did not interfere with curli and pEtN-cellulose production, since colonies of reduced size were still wrinkled as is particularly visible in the absence of the large amount of viscous colanic acid with strain AR155 (Figure 3).

### *Ascaris suum* ESP possess agglutinating activity

**Figure 4:**
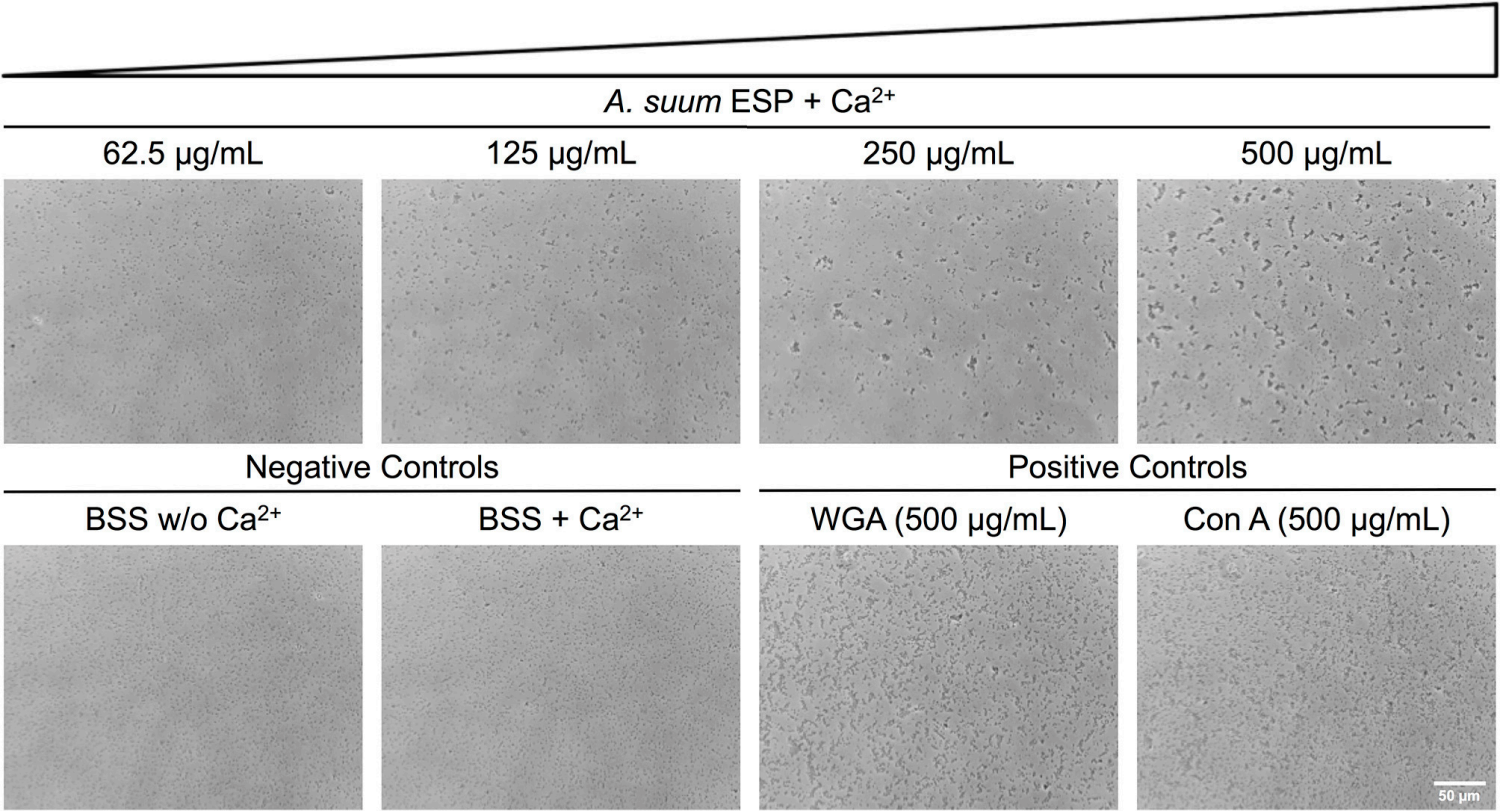
*Ascaris suum* excretory/secretory products cause bacterial agglutination. Bacterial agglutination in the presence of adult *A. suum* ESP and 10 mM CaCl_2_. Representative images of agglutination of *E. coli* IMT19224 with serial dilutions (1/2 factor) of *A. suum* ESP. Controls of agglutination include adult worm media (BSS) with and without CaCl_2_ as well as the C-type lectins wheat germ agglutinin (WGA) and concanavalin A (Con A). Bacteria visualized at 400X magnification.

Having demonstrated growth-inhibiting and biofilm-disrupting capabilities of *A. suum* ESP, we sought to determine if the nematodes could defend themselves against microbial threats without overtly killing bacteria. In addition to the inhibition of bacterial growth in the radial diffusion and macrocolony assays, we observed that also in our submerged biofilm assays some bacteria were still able to survive the treatment and reasoned that there may be non-lethal defense mechanisms employed by the worms such as neutralization via agglutination. In order to test the agglutinating activity of *A. suum* ESP, we treated *E. coli* ST131 IMT19224 with adult *A. suum* ESP (1 mg/mL) in the presence and absence of CaCl_2_ (10 mM) and observed calcium-dependent agglutinating activity (Figure 4). The calcium-dependence implies the activity of C-type lectin domain-containing (CTLD) proteins which require calcium in order to exert their agglutinating and glycan-binding activities (Mayer et al., 2017). Similar results were obtained for the biofilm-forming *E. coli* K12 AR3110 and AR115 strains (Supplementary Figures 1, 2).Thus, in addition to inhibiting bacterial growth and disrupting bacterial biofilm formation, *A. suum* adult ESP are also capable of neutralizing infectious threats by agglutinating bacteria.

### *Ascaris suum* ESP and body fluid contain proteins and peptides with known and predicted antimicrobial activities

In order to characterize ESP and body fluid of *A. suum* with respect to defense strategies that the nematode may employ in its microbial environment, we used native nematode material and omitted ultrafiltration-based concentration and trichloroacetic acid-mediated precipitation steps during our sample preparation which would have removed key antimicrobial components such as antimicrobial peptides from the final sample. By LC-MS/MS analysis, we assessed the protein and peptide constituents of ESP from different larval stages, including *in vitro*-hatched L3, lung-stage L3, intestinal-stage L4, and from adults, as well as BF obtained from adult males. The analysis revealed the presence of several proteins and peptides with known and predicted roles in nematode defense (Table 1), including galectins, C-type lectin domain-containing (CTLD) proteins, AMPs, a lysozyme (GH family 25 lysozyme 2), and a cysteine protease inhibitor (cystatin). Adult male and female ESP did not seem to differ in antimicrobial contents and are therefore shown together. Interestingly, we detected all of the aforementioned antimicrobial proteins and peptides in the ESP of adult nematodes, whereas we detected none of the proteins of interest in the ESP of lung-stage L3 larvae. ASABF-alpha, -beta, and –epsilon were detected only in adult ESP. In contrast, members of the cecropin family were detected in adult ESP and body fluid as well as in L4-larval ESP as well as cecropin P1 or P2 *in vitro*-hatched L3 ESP. While a significant and distinct peptide was detected and attributed to cecropin P1 in adult, L4, and *in vitro*-hatched L3 ESP as well as in BF, the same peptide could be attributed to cecropin P2; therefore, it is unclear if only cecropin P2 was detected or both cecropin P1 and P2. Cecropin P3 was detected in adult ESP and BF, as well as L4-stage ESP while cecropin P4 was detected only in adult ESP and BF, but not in larval material. The aforementioned lysozyme was detected only in adult ESP. Lectins, including CTLD proteins and galectins, were detected only in adult ESP but not in adult body fluid or in larval material. We detected seven unique CTLD proteins (including the three uncharacterized proteins, all of which contain CTLDs), though two of the seven did not contain signal peptides. Similarly, both galectins detected also did not contain signal peptides and were not predicted to be non-classically secreted using SecretomeP. Cystatin was detected in adult and *in vitro-*hatched L3 ESP. Cystatins from chickens and humans possess antibacterial activity (Blankenvoorde et al., 1998; Wesierska et al., 2005; Ganeshnarayan et al., 2012) whereas helminth cystatins, including from *Ascaris*, have a well-established role in modulating host immunity (Hartmann and Lucius, 2003; Mei et al., 2014; Coronado et al., 2017). Whether nematodes use cystatins to modulate the gut microbiota in addition to host immune cells requires further study. The results of the LC-MS/MS analysis demonstrate that *A. suum* secretes diverse antimicrobial proteins and peptides which explain the various antibacterial activities we have observed. Furthermore, these factors likely act together to shape the nematode's microbial environment within the intestine of its host.

**Table 1:** Proteins and peptides with known and predicted antimicrobial activities detected in excreted/secreted products and body fluid of *A. suum*^a^.

**Protein name uniprot^b^**	**Protein mass (Da^b^)**	**Signal peptide^c^**	**Accession number uniprot**
C-type lectin domain-containing protein 160	41,886	+	F1L7R9
C-type lectin domain-containing protein 160	47,612	+	F1L4K4
C-type lectin domain-containing protein 160	43,174		F1L8I9
C-type lectin protein 160	60,173	−	F1L0R7
32 kDa beta-galactoside-binding lectin	32,483	−	F1L893
32 kDa beta-galactoside-binding lectin	31,791	−	F1LAD2
GH family 25 lysozyme 2	24,644	+	F1LE63
GH family 25 lysozyme 2	21,687	−	F1LEA7
Cystatin	13,961	+	F1LHQ3
ASABF-alpha	9,843	+	P90683
ASABF-beta	9,219	+	Q8MMG8
ASABF-epsilon	7,037	+	Q8IAC9
Cecropin-P1	7,876	+	P14661
Cecropin-P2	9,760	+	Q5H7N6
Cecropin-P3	8,381	+	Q5H7N5
Cecropin-P4	8,424	+	Q5H7N4
**ADULT MALE BODY FLUID**			
Cecropin-P1 and/or Cecropin-P2	7,876/9,760	+	P14661/Q5H7N6
Cecropin-P3	8,381	+	Q5H7N5
Cecropin-P4	8,424	+	Q5H7N4
**L4-STAGE LARVAE**			
Cecropin P1	7,876	+	P14661
Cecropin-P2	9,760	+	F1LBL1
Cecropin-P3	8,381	+	Q5H7N5
***IN VITRO*****-HATCHED L3 LARVAE**			
Cecropin-P1 or Cecropin-P2	7,876	+	P14661/Q5H7N6
Cystatin	13,961	+	F1LHQ3

a *Extended version of table available in Supplementary Material*.

b *Protein name and mass from Uniprot database (https://www.uniprot.org)*.

c *Identified proteins predicted to contain secretory signal peptide (+) or not (−) using SignalP*.

## Discussion

Intestinal parasites inhabit a microbe-rich environment. Diverse interactions between environmental microbes and free-living nematodes have been described, and similarly, the microbes in the host-gut may present benefits and risks for parasitic nematodes as they establish themselves in the niche of the intestine and migrate through the host tissue without eliciting overt inflammation. How *A. suum* survives in the small intestine of its porcine host has thus far been studied with a focus on host-pathogen interactions, whereas the interactions between *Ascaris* and the host-gut microbiota remain largely unexplored. Secreted products of helminths play various roles during the establishment of nematode infections, including invasion, migration, immune avoidance and immune modulation (Coakley et al., 2016). Hence, examining the role of secreted nematode products in nematode-microbe interactions is necessary to gain insights into the intricate trilateral interplay between the parasite, the host and the intestinal microbes during *A. suum* infection.

In this study, we demonstrated that *A. suum* ESP from different life stages possess antimicrobial activity against gram-negative and gram-positive bacteria (Figure 1). Interestingly, detectable antibacterial activity was limited to samples obtained from intestine-dwelling life stages, namely ESP from fourth larval-stage and adult worms, as well as body fluid from adult worms. Several proteins and peptides with known and predicted roles in antimicrobial defense were detected in these *A. suum* ESPs. In the nematode secreted products and BF samples, we detected members of the ASABF and cecropin AMP families (Table 1), previously shown to possess broad-spectrum antimicrobial activity (Pillai et al., 2003, 2005), accounting for observed antibacterial activities. Adult ESP also contained the highest diversity of potential antimicrobial components, including lectins, cystatin, and a lysozyme, GH family 25 lysozyme 2. To our knowledge, antibacterial activities of *Ascaris* lectins, cystatin, and lysozyme have not been reported previously; however, adult female BF has been reported to possess lysozyme-like and agglutinating activities, though specific factors were not identified (Kato, 1995). We were unable to detect antibacterial activity of ESP from *in vitro*-hatched L3 larvae and from lung-stage L3 larvae. Third-stage larvae hatch from infectious eggs protected by the L2 cuticle before migrating through host tissues (Douvres et al., 1969). As the liver is continuously exposed to microbial antigens from the gut, hepatic immune cells are particularly primed to deal with incoming threats (McNamara and Cockburn, 2016). L3-stage larvae may therefore be protected from microbial threats by cuticle barriers for the few hours in the intestine before entering the host, and by the host-antimicrobial immune system responding to any microbes that may be carried with the larvae as they penetrate through the intestinal tissue to the liver. While microbial threats are abundant in the intestine, tissue migration presents other unique challenges for the nematode larvae. Cystatin likely plays an important role in the interaction between migratory *A. suum* larvae and host immune cells; if it possesses antimicrobial activity as shown for cystatins from chickens and humans (Blankenvoorde et al., 1998; Wesierska et al., 2005; Ganeshnarayan et al., 2012) remains to be determined. Thus, our data indicate tissue migratory third-stage larvae may not produce high quantities of antimicrobials. In contrast, we detected considerable antibacterial activity in cecropin-containing ESP from L4-stage larvae which have undergone further development after re-entering the intestine and thereby facing the presence of the intestinal microbiota. Furthermore, material harvested from adult nematodes, which have to contend with the host microbiota for the majority of the worm's lifespan, also showed considerable antibacterial activity. To counteract a diversity of potential threats originating from the microbiota, *Ascaris* is armed with several antimicrobial factors resulting in broad-spectrum antibacterial activity.

Studies in *C. elegans* have demonstrated the importance of biofilms in bacterial-nematode interactions. Within biofilms, bacteria are bound together within an extracellular matrix composed of exopolysaccharides, proteins, and nucleic acids (Hall-Stoodley et al., 2004) which provides support and protection, allowing bacteria to withstand higher concentrations of antibiotics (Dufour et al., 2010). Biofilm exopolysaccharides have been shown to enhance virulence of *S. epidermidis* during colonization of the *C. elegans* intestine in addition to enhancing bacterial resistance to nematode antimicrobial factors (Begun et al., 2007). Interestingly, biofilm forming *B. subtilis* promote oxidative stress resistance, thermotolerance, and upregulated expression of a lysozyme leading to enhanced resistance to worm killing by the pathogenic *Pseudomonas aeruginosa* (Smolentseva et al., 2017). Though the experimental settings differ, these studies highlight the importance of the biofilm lifestyle to nematode health. Thus, as biofilms might also influence parasitic nematode physiology, we studied the impact of *Ascaris* ESP on biofilm formation by *E. coli* K-12 strain AR3110. ESP from adult worms clearly resulted in a dose-dependent reduction in biomass accumulation in the submerged biofilm model (Figure 2). *E. coli* AR3110 also form macrocolony biofilms with pEtN-cellulose and amyloid curli fibers as key components of the extracellular matrix (Serra et al., 2013; Thongsomboon et al., 2018). In the presence of adult *A. suum* ESP, macrocolony formation was considerably disrupted and was accompanied by the production of the complex exopolysaccharide colanic acid, while the production of pEtN-cellulose and curli fibers (reflected by colony wrinkling) was not affected (Figure 3). Colanic acid production, which is under the control of the RcsC/RcsB phosphorelay cascade (Majdalani et al., 2005) and is induced in response to cell envelope stress (Laubacher and Ades, 2008), confers resistance to antimicrobial peptides (Detweiler et al., 2003). Hence, nematode antimicrobial factors present in the *A. suum* ESP, especially AMPs, represent extracytoplasmic stress and, by inducing production of colanic acid, modify bacterial biofilm formation. While the inability of *E. coli* to produce colanic acid increased the growth inhibitory effects of *A. suum* ESP, a portion of the bacterial population was still able to survive the treatment (Figure 3). Similarly, resistance to AMPs allows *S. typhimurium* to persist in the intestine of *C. elegans* (Alegado and Tan, 2008); while nematodes release factors to defend themselves against bacterial threats, some can withstand these assaults. While bacteria are able to colonize the intestine of *Ascaris*, as determined by culture-based methods (Nalin and McLaughlin, 1976; Hsu et al., 1986; Shahkolahi and Donahue, 1993), the role of biofilms in microbial colonization of *A. suum* and interplay with the host microbiota during ascariasis require further study.

In addition to bactericidal factors such as ASABFs and Cecropins, we also detected lectins, including C-type lectin domain-containing (CTLD) proteins and galectins (Table 1). CTLD proteins recognize and bind to carbohydrate ligands and are critical in immunity (Brown et al., 2018). CTLD proteins can be transmembrane proteins, functioning as cell surface receptors, or can be secreted. A previous study isolated three CTLD proteins from the murine intestinal nematodes *Heligmosomoides polygyrus* and *Nippostrongylus brasiliensis* (Harcus et al., 2009). The authors reported that these lectins are primarily expressed in the intestine-dwelling adult stages; however, bacterial binding functions were not assessed in their study. *C. elegans* possesses an estimated 283 CTLD (*clec*) genes, the majority of which are thought to be secreted (Pees et al., 2016). Previous studies have demonstrated that during infection with *S. marcescens, clec-39*,−*49*, and *-50* are upregulated and worms deficient in *clec-39* are more susceptible to infection with *S. marcescens* (Mallo et al., 2002; Engelmann et al., 2011; Miltsch et al., 2014). Additionally, recombinant CLEC-39 and−49 were shown to bind *S. marcescens* without killing the bacteria (Miltsch et al., 2014). We demonstrated calcium-dependent agglutinating activity of adult *A. suum* ESP (Figure 4), likely due to the CTLD proteins we detected. Both mammalian and non-mammalian hosts use lectins to shape the intestinal microbiota (Pang et al., 2016), an effect which could be compounded by secreted *A. suum* CTLD proteins. Galectins are β-galactoside-binding proteins also thought to function in host defense (Vasta, 2009). *C. elegans* deficient in the galectin LEC-8 were more susceptible to infection with *Bacillus thuringiensis* (Ideo et al., 2009). Interestingly, galectins do not typically contain secretory signal peptides but many localize extracellularly and are thought to be non-classically secreted (Barondes et al., 1994; Hughes, 1999). The galectins reported in our study were not predicted to contain signal peptides or to be secreted through non-classical pathways. However, though their presence in the *A. suum* ESP may contribute to agglutinating activity, their roles in nematode defense and in shaping the porcine intestinal microbiota need further investigation.

In this study we described diverse impacts of *A. suum* ESP on bacterial species from direct antimicrobial activity, disrupted biofilm formation, and neutralization by agglutination. These observations correlated with proteins and peptides detected in the ESP by mass spectrometry analysis and suggest that intestinal nematodes employ multiple strategies in their interactions with bacteria. Studies in infection models of *C. elegans* reveal pathogen and tissue-specific gene expression changes (Engelmann et al., 2011) along with differentially synthesized proteins in response to different microbial pathogens (Bogaerts et al., 2010a,b). These studies identified a diversity of upregulated factors including antimicrobial peptides, lectins, and lysozymes, all of which we detected in *A. suum* ESP. These multiple factors would then act in concert with one another to endow nematodes with a broad-spectrum defense system to allow survival in a microbial environment, as faced by *A. suum* in the porcine intestine. While we focused on the protein components of *Ascaris* ESP, it is important to note that helminth ESP also contain RNAs (Buck et al., 2014) and metabolites such as short-chain fatty acids (Zaiss et al., 2015) which in addition to modulating host immunity, may also impact the microbiota. Further study is required to determine the role of non-protein contents in shaping the microbiota; however, antibacterial activity described in our study due to combination effects of the various constituents of *A. suum* ESP have been accounted for by our use of native material.

In summary, our findings suggest that intestine-dwelling life stages of *A. suum* employ diverse antimicrobial strategies to establish themselves amongst the host microbiota. Our results provide a first indication of the direct impact of an intestinal nematode on its immediate microbial environment. Furthermore, our results suggest that the antimicrobial potential of nematode products differ depending on the parasite life-stage and corresponding host-environments. While metabolic and host immune factors would also contribute to an altered microbiome during helminth infection, we propose that nematodes themselves also have a direct role in shaping the microbiota as they establish themselves in the host gut, involving the secreted products and antimicrobial activities described herein. These changes would be more pronounced with a high worm burden as the local concentration of nematode antimicrobials would likely be higher. The defense strategies discussed in this study involve killing and non-killing mechanisms exerted by several different secreted factors acting in combination, as exemplified by the constitution and diverse activities of *A. suum* ESP. Together, these factors allow nematodes to carve out a niche to survive within a microbial environment and while doing so, may be partially responsible for changes to the intestinal microbiome during helminth infection.

## Author contributions

All authors gave final approval for manuscript publication. Project designed by AM, SH, and JS. Microbiological experiments designed by AM, SG, RH, and DS. Mass spectrometry analysis performed by KJ and AN. Peptides for mass spectrometry analysis synthesized by PH. AM performed all experiments. All authors interpreted data. Manuscript was written by AM and SH with input from the other authors.

### Conflict of interest statement

The authors declare that the research was conducted in the absence of any commercial or financial relationships that could be construed as a potential conflict of interest.
