# Corrigendum: The Intestinal Roundworm *Ascaris suum* Releases Antimicrobial Factors Which Interfere With Bacterial Growth and Biofilm Formation

**DOI:** 10.3389/fcimb.2018.00367

**Published:** 2018-10-16

**Authors:** Ankur Midha, Katharina Janek, Agathe Niewienda, Petra Henklein, Sebastian Guenther, Diego O. Serra, Josephine Schlosser, Regine Hengge, Susanne Hartmann

**Affiliations:** ^1^Department of Veterinary Medicine, Institute of Immunology, Freie Universität Berlin, Berlin, Germany; ^2^Charité – Universitätsmedizin Berlin, Corporate Member of Freie Universität Berlin, Humboldt-Universität zu Berlin, and Berlin Institute of Health, Institute of Biochemistry, Shared Facility for Mass Spectrometry, Berlin, Germany; ^3^Charité – Universitätsmedizin Berlin, Corporate Member of Freie Universität Berlin, Humboldt-Universität zu Berlin, and Berlin Institute of Health, Institute of Biochemistry, Berlin, Germany; ^4^Department of Veterinary Medicine, Institute of Animal Hygiene and Environmental Health, Freie Universität Berlin, Berlin, Germany; ^5^Department of Pharmaceutical Biology, Institute of Pharmacy, Ernst-Moritz-Arndt-Universität Greifswald, Greifswald, Germany; ^6^Institute of Biology/Microbiology, Humboldt-Universität-zu-Berlin, Berlin, Germany

**Keywords:** intestinal nematode, ascariasis, helminth, microbiota, antimicrobial peptides, biofilm, lectin

In the published article, there was an error in affiliations 2 and 3. We incorrectly stated they were: 2. Shared Facility for Mass Spectrometry, Berlin Institute of Health, Institute of Biochemistry, Charité – Universitätsmedizin Berlin, Corporate Member of Freie Universität Berlin, Humboldt-Universität zu Berlin, Berlin, Germany

3. Berlin Institute of Health, Institute of Biochemistry, Charité – Universitätsmedizin Berlin, Corporate Member of Freie Universität Berlin, Humboldt-Universität zu Berlin, Berlin, Germany

The correct affiliations appear in the author list above. The authors apologize for this error and state that this does not change the scientific conclusions of the article in any way.

The original article has been updated.

## Conflict of interest statement

The authors declare that the research was conducted in the absence of any commercial or financial relationships that could be construed as a potential conflict of interest.

