# Supplementary material for: The Intestinal Roundworm Ascaris suum Releases Antimicrobial Factors Which Interfere With Bacterial Growth and Biofilm Formation
Source: Front Cell Infect Microbiol. 2018 Aug 7;8:271. doi: 10.3389/fcimb.2018.00271 (PMC6090379; doi:10.3389/fcimb.2018.00271)
Supplement: Supplementary file 1 [file Data_Sheet_1.docx]

Supplementary Material

The intestinal roundworm *Ascaris suum* releases antimicrobial factors which interfere with bacterial growth and biofilm formation

Ankur Midha^1^, Katharina Janek^2^, Agathe Niewienda^2^, Petra Henklein^3^, Sebastian Guenther^4,5^ Diego O. Serra^6^, Josephine Schlosser^1^, Regine Hengge^6^, Susanne Hartmann^1*^

^1^Institute of Immunology, Department of Veterinary Medicine, Freie Universität Berlin, Berlin, Germany

^2^Charité – Universitätsmedizin Berlin, corporate member of Freie Universität Berlin, Humboldt-Universität zu Berlin, and Berlin Institute of Health, Institute of Biochemistry, Shared Facility for Mass Spectrometry, Chariteplatz 1, D-10117 Berlin, Germany

^3^Charité – Universitätsmedizin Berlin, corporate member of Freie Universität Berlin, Humboldt-Universität zu Berlin, and Berlin Institute of Health, Institute of Biochemistry, Chariteplatz 1, D-10117 Berlin, Germany

^4^Institute of Animal Hygiene and Environmental Health, Department of Veterinary Medicine, Freie Universität Berlin, Berlin, Germany

^5^Institute of Pharmacy, Department of Pharmaceutical Biology, Ernst-Moritz-Arndt-Universität Greifswald, Greifswald, Germany

^6^Institute of Biology / Microbiology, Humboldt-Universität-zu-Berlin, Berlin, Germany

*** Correspondence:**Susanne Hartmann
susanne.hartmann@fu-berlin.de

## Supplementary Figures and Tables

**Supplementary Figure 1. *Ascaris suum* excretory/secretory products cause bacterial agglutination of biofilm-forming *E. coli* AR3110.**

Bacterial agglutination in the presence of adult *A. suum* ESP and 10 mM CaCl_2_. Representative images of agglutination of *E. coli* K12 AR3110 with serial dilutions (1/2 factor) of *A. suum* ESP. Controls of agglutination include adult worm media (BSS) with and without CaCl_2_ as well as the C-type lectin concavalin A. Bacteria visualized at 400X magnification.

**Supplementary Figure 2. *Ascaris suum* excretory/secretory products cause bacterial agglutination of biofilm-forming *E. coli* AR155.**

Bacterial agglutination in the presence of adult *A. suum* ESP and 10 mM CaCl_2_. Representative images of agglutination of *E. coli* K12 AR155 with serial dilutions (1/2 factor) of *A. suum* ESP. Controls of agglutination include adult worm media (BSS) with and without CaCl_2_ as well as the C-type lectin concavalin A. Bacteria visualized at 400X magnification.

**Supplementary Table 1. Proteins and peptides with known and predicted antimicrobial activities detected in excreted/secreted products and body fluid of *A. suum.***

^a^https://www.uniprot.org

^b^Identified proteins predicted to contain secretory signal peptide (+) or not (-) using SignalP.

^c^The proteins were identified by mass spectrometry with accession numbers from the databases: 1, http://nematode.net/NN3_frontpage.cgi?navbar_selection=speciestable&subnav_selection=Ascaris_suum; 2, http://aps.unmc.edu/AP/main.php; 3, Swissprot 4, Uniprot *Ascaris suum*

^d^Mascot

^†^Closest match: uncharacterized protein (*Toxocara canis*) containing two C-type lectin domains

^§^Identity was confirmed by comparison of the MS/MS spectrum with the fragmentation pattern of a synthetic reference peptide.

^#^same sequence for Cecropin P1 and P2

^*^p<0.00001

| **Protein Characteristics** 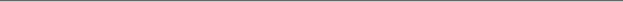 | | | | **Identification by Mass Spectrometry** 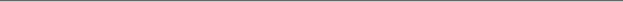 | | | | | | |
| --- | --- | --- | --- | --- | --- | --- | --- | --- | --- | --- |
| **Protein Name Uniprot^a^** | **Protein Mass (Da) Uniprot** | **SP^b^** | **Accession Number Uniprot** | **Accession Number of Identified Protein** | **Database^c^** | **Protein Mass (Da)** | **Protein Score^d^** | **Number of significant distinct sequences (p<0.01)** | **Number of unique sequences** | **Sequence Coverage** |
|  |  |  |  |  |  |  |  |  |  |  |
| **Adult, male or female** |  |  |  |  |  |  |  |  |  |  |
| uncharacterized protein | 51771 | - | A0A183VED4^†^ | AS00034 | 1 | 23257 | 87 | 3 | 3 | 18 |
| C-type lectin domain-containing protein 160 | 41886 | + | F1L7R9 (aa 1-364) | AS03243 | 1 | 41584 | 768 | 14 | 8 | 86 |
| C-type lectin domain-containing protein 160 | 47612 | + | F1L4K4 (aa 31-259) | AS02827 | 1 | 30453 | 787 | 8 | 7 | 45 |
| C-type lectin domain-containing protein 160 | 43174 | + | F1L8I9 (aa 13-397) | AS01800 | 1 | 24619 | 335 | 7 | 7 | 46 |
| uncharacterized protein | 85734 | + | A0A0M3HP70 | AS02732 | 1 | 32062 | 357 | 7 | 7 | 33 |
| C-type lectin protein 160 | 60173 | - | F1L0R7 (aa 139-302) | AS04212 | 1 | 18321 | 338 | 4 | 2 | 34 |
|  |  |  | F1L0R7 (aa 227-430) | AS10343 | 1 | 23961 | 768 | 14 | 8 | 86 |
|  |  |  | F1L0R7 (aa 348-531) | AS02748 | 1 | 22787 | 464 | 10 | 7 | 65 |
| 32 kDa beta-galactoside-binding lectin | 32483 | - | F1L893 | F1L893 | 2 | 32483 | 385 | 17 | 17 | 57 |
| 32 kDa beta-galactoside-binding lectin | 31791 | - | F1LAD2 | F1LAD2 | 2 | 31791 | 546 | 12 | 11 | 53 |
| GH family 25 lysozyme 2 | 24644 | + | F1LE63 | AS00167 | 1 | 23880 | 567 | 8 | 8 | 37 |
| GH family 25 lysozyme 2 | 21687 | - | F1LEA7 | AS00467 | 1 | 10338 | 94 | 5 | 4 | 38 |
| uncharacterized protein | 19086 | + | A0A0M3HT95 | AS00263 | 1 | 17357 | 471 | 4 | 4 | 45 |
| Cystatin | 13961 | + | F1LHQ3 | AS02342 | 1 | 14281 | 343 | 5 | 5 | 50 |
| ASABF-alpha | 9843 | + | P90683 | AP01523 | 3 | 8408 | 121 | 5 | 4 | 37 |
| ASABF-beta | 9219 | + | Q8MMG8 | AS01881 | 1 | 10403 | 52 | 2 | 1^§^ | 20 |
| ASABF-epsilon | 7037 | + | Q8IAC9 | AS02651 | 1 | 7457 | 78 | 2 | 2 | 38 |
| Cecropin-P1 | 7876 | + | P14661 | P14661 | 4 | 7876 | 71 | 1^#*^ | 0 | 20 |
| Cecropin-P2 | 9760 | + | Q5H7N6 | Q5H7N6 | 4 | 9760 | 96 | 2 | 1^§^ | 36 |
| Cecropin-P3 | 8381 | + | Q5H7N5 | Q5H7N5 | 4 | 8381 | 68 | 2 | 2 | 35 |
| Cecropin-P4 | 8424 | + | Q5H7N4 | Q5H7N4 | 4 | 8424 | 42 | 1 | 1^§^ | 18 |
|  |  |  |  |  |  |  |  |  |  |  |
| **Adult male body fluid** |  |  |  |  |  |  |  |  |  |  |
| Cecropin-P1 and/or  Cecropin-P2 | 7876 / 9760 | + | P14661 / Q5H7N6 | P14661 / Q5H7N6 | 4 | 7876 / 9760 | 54 | 1^#*^ | 0 | 20 |
| Cecropin-P3 | 8381 | + | Q5H7N5 | CECP3_ASCSU | 4 | 8376 | 29 | 1 | 1^§^ | 14 |
| Cecropin-P4 | 8424 | + | Q5H7N4 | CECP4_ASCSU | 4 | 8418 | 30 | 1 | 1^§^ | 19 |
|  |  |  |  |  |  |  |  |  |  |  |
| **L4-stage larvae** |  |  |  |  |  |  |  |  |  |  |
| Cecropin P1 | 7876 | + | P14661 | P14661 | 4 | 7871 | 66 | 1^#*^ | 0 | 20 |
| Cecropin-P2 | 9760 | + | F1LBL1 | F1LBL1_ASCSU | 4 | 9760 | 71 | 2 | 1^§^ | 36 |
| Cecropin-P3 | 8381 | + | Q5H7N5 | Q5H7N5 | 4 | 8381 | 78 | 1 | 1^§^ | 22 |
|  |  |  |  |  |  |  |  |  |  |  |
| ***in vitro*-hatched L3 larvae** |  |  |  |  |  |  |  |  |  |  |
| Cecropin-P1 or Cecropin-P2 | 7876 | + | P14661 / Q5H7N6 | P14661 / Q5H7N6 | 4 | 7871 | 41 | 1^#*^ | 0 | 20 |
| Cystatin | 13961 | + | F1LHQ3 | AS02342 | 1 | 14281 | 149 | 3 | 3 | 28 |
|  |  |  |  |  |  |  |  |  |  |  |
